# Non-invasive Photodynamic Therapy in Brain Cancer by Use of Tb^3+^-Doped LaF_3_ Nanoparticles in Combination with Photosensitizer Through X-ray Irradiation: A Proof-of-Concept Study

**DOI:** 10.1186/s11671-017-1840-3

**Published:** 2017-01-21

**Authors:** Min-Hua Chen, Yi-Jhen Jenh, Sheng-Kai Wu, Yo-Shen Chen, Nobutaka Hanagata, Feng-Huei Lin

**Affiliations:** 1 Institute of Biomedical Engineering, National Taiwan University, Taipei, 10051 Taiwan; 2 Nanotechnology Innovation Station, National Institute for Materials Science, Tsukuba, Ibaraki, 3050047 Japan; 3 Graduate School of Life Science, Hokkaido University, Sapporo, Hokkaido, 0600808 Japan; 4 Institute of Biomedical Engineering and Nanomedicine, National Health Research Institutes, Miaoli County, 35053 Taiwan

**Keywords:** Non-invasive, Photodynamic therapy, LaF_3_:Tb, X-ray, Brain cancer

## Abstract

The use of photodynamic therapy (PDT) in the treatment of brain cancer has produced exciting results in clinical trials over the past decade. PDT is based on the concept that a photosensitizer exposed to a specific light wavelength produces the predominant cytotoxic agent, to destroy tumor cells. However, delivering an efficient light source to the brain tumor site is still a challenge. The light source should be delivered by placing external optical fibers into the brain at the time of surgical debulking of the tumor. Consequently, there exists the need for a minimally invasive treatment for brain cancer PDT. In this study, we investigated an attractive non-invasive option on glioma cell line by using Tb^3+^-doped LaF_3_ scintillating nanoparticles (LaF_3_:Tb) in combination with photosensitizer, meso-tetra(4-carboxyphenyl)porphyrin (MTCP), followed by activation with soft X-ray (80 kVp). Scintillating LaF_3_:Tb nanoparticles, with sizes of approximately 25 nm, were fabricated. The particles have a good dispersibility in aqueous solution and possess high biocompatibility. However, significant cytotoxicity was observed in the glioma cells while the LaF_3_:Tb nanoparticles with MTCP were exposed under X-ray irradiation. The study has demonstrated a proof of concept as a non-invasive way to treat brain cancer in the future.

## Background

Malignant gliomas are the most common type of primary brain tumors; the survival rate is about 2 years for patients with grade III tumors and 1 year for those with grade IV tumors, and the average life expectancy at 5 years is not higher than 5% in Taiwan [1, 2]. Because malignant gliomas are located in billions of interacted neurons and physiologic barriers, especially the blood-brain barrier (BBB), which protects infiltrating glioma cells from the effect of chemotherapeutic agents, this causes gliomas to be difficult to treat. Despite the advances in conventional approaches, including surgery, radiotherapy, and chemotherapy, the effectiveness of treatment in these patients remains limited. Many of the current treatments in malignant gliomas have inadequate drug delivery and cause damage to healthy brain tissue [3].

Photodynamic therapy (PDT) is based on the concept of proceeding through the activation of photosensitizer by a specific light wavelength (620–690 nm) to produce the predominant cytotoxic agent, such as free radicals and singlet oxygen (^1^O_2_). The use of PDT in the treatment of brain tumors has produced exciting results in clinical trials over the past decade [4]. PDT is expected to be the breakthrough for the treatment of malignant glioma because it has selective cytotoxicity to target infiltrating malignant brain tumor cells and induces a cytotoxic reaction only in the light-exposed areas. Nevertheless, the limited penetration range of light causes the assessment of the light distribution and tumoricidal effects of PDT inside the brain to be difficult [5, 6]. To ensure adequate dispersion of light to the area of brain tumors, two strategies that use fiber optic devices could be carried out, the usage of which is determined by the size, stage, and localization of tumor. First, interstitial PDT is a method by stereotactically inserting optical fibers and filling the tumor cavity with a light-diffusing medium, such as lipid solution, to spread the light evenly throughout the tumor cavity. Second, an intraoperative of the balloon irradiator in a resected tumor cavity after an invasive craniotomy could be used [4, 7]. However, all of these treatments require the external optical fibers be placed within the brain tumors. Consequently, there exists the need for a minimally invasive brain cancer PDT.

An attractive non-invasive option is to use scintillating nanoparticles with photosensitizer through X-ray irradiation to enable the light source to reach a higher tissue penetration depth in the range of 8–14 cm [6]. This approach is based on the concept that scintillating nanoparticles, such as Tb^3+^-doped LaF_3_ crystal (LaF_3_:Tb), can locally convert X-ray into light and the emitted luminescences are able to activate the photosensitizers on the mechanism of fluorescence resonance energy transfer (FRET), further resulting in activating photosensitizer to induce ^1^O_2_ for cancer therapy [8]. The conversion of X-ray into fluorescence emission by LaF_3_:Tb is based on the mechanism that Tb^3+^ ions exhibit the transitions resulting mainly from the excited level, ^5^D_4_, down to the lower levels, ^7^F_j_ (*j* = 6–3), and can be accompanied by the photoluminescence properties as Tb^3+^ doped in low vibrational energy and high resistivity properties of LaF_3_ host material [9, 10]. LaF_3_:Tb has demonstrated luminescence at 487, 542, 582, and 620 nm under the excitation of X-ray [10]. Upon X-ray irradiation, photosensitizers are activated by photons emitted from LaF_3_:Tb nanoparticles while the absorption band of photosensitizers and the emission band of scintillation nanoparticles overlap. It has been reported that approximately 56.7% of energy can be transferred from X-ray to the adjacent photosensitizers via LaF_3_:Tb nanoparticles [11].

X-ray-excited PDT, based on scintillating nanoparticles, was first introduced by Chen and Zhang [12] in 2006 and recently several studies have demonstrated this effect into proof of concept [8, 13–15]. For future clinical applications, the photosensitizers can be loaded onto nanoparticles, which can lead to a more direct and specific localization of the photosensitizer to the brain tumor sites and increase the efficiency and selectivity in treatment. Another aspect of this approach is the treatments using nanoparticles are regarded as one of the most promising approaches to transport photosensitizers across the barriers of BBB as well as in combination of PDT with radiotherapy for brain cancer treatment [16, 17]. More importantly, X-ray not only can penetrate the tissue much deeper than the laser light source but also can extend the popularity of PDT to resource-limited hospitals because the X-ray system is widely used in the clinic for both diagnosis and therapy.

Here we demonstrate a proof of concept as a non-invasive PDT on glioma cell line (9L) by the treatment of soft X-ray (180 kVp) and photosensitizer, meso-tetra(4-carboxyphenyl)porphyrin (MTCP), employing scintillating nanoparticles. Although scintillating nanoparticles have been studied in PDT [13, 15, 18], to the best of the authors’ knowledge, the non-invasive PDT concept of using scintillating nanoparticles in brain cancer cells has not been described.

## Methods

### Synthesis of LaF_3_:Tb Nanoparticles

The aqueous-dispersible LaF_3_:Tb nanoparticles were synthesized by a modified wet chemical precipitation method according to Liu et al. [10]. Three major components, La(NO_3_)_3_·6H_2_O, TbCl_3_·6H_2_O, and NH_4_F solutions, were purchased from Sigma-Aldrich. Briefly, 4.3 mmol La(NO_3_)_3_·6H_2_O and 1.1 mmol TbCl_3_·6H_2_O were dissolved in 150 ml of de-ionized water, followed by 58.4 mmol of NH_4_F solution with a volume of 46 ml added dropwise to the complex solution. The reaction was stirred for 2 h at room temperature. Finally, the ultimate solution was centrifuged, washed with de-ionized water three times, and stored at 4 °C until use.

### Characterization of LaF_3_:Tb Nanoparticles

The morphology of particles was observed by dropping onto a copper grid using a transmission electron microscopy (TEM; Hitachi H-7100, Japan). Energy-dispersive X-ray spectroscopy (EDX) system attached to TEM was used to analyze the composition of ions in particles. X-ray diffraction (XRD; Geiger Flex, Rigaku) was utilized to identify the crystalline phase composition using Cu Kα radiation (*λ* = 0.15406 nm) with the potential at 30 kV and the current at 20 mA. The lattice parameters (*a*-axis and *c*-axis) were calculated from the major reflection peaks, (111), (300), (113), and (302), with the equation in the hexagonal crystal system: 1/*d* ^2^ = 4/3{(*h* ^2^ + *hk* + *k* ^2^)/*a* ^2^} + (*l* ^2^/*c* ^2^), where *h*, *k*, and *l* are Miller’s indices and *d* is the interplanar spacing [19]. The fluorescence emission characteristics of LaF_3_:Tb were measured using the fluorescence spectrometer (F-7000 FL, Hitachi) with excitation at 260 nm.

### Cell Viability

The viability of LaF_3_:Tb particles on the fibroblast cell line (3T3) was evaluated by cell proliferation reagent (WST-1, Roche). 3T3 cells cultivated in Dulbecco’s modified Eagle’s medium with high glucose (DMEM, Sigma-Aldrich) supplemented with 10% fetal bovine serum (FBS) were seeded in a 96-well petri dish (3000 cells/well) and kept in a humidified environment with 5% CO_2_ at 37 °C overnight. Then, cells were exposed to different concentrations of LaF_3_:Tb particles, followed by 4 h of incubation period. Later, the media was replaced with fresh media and further incubated for another 24 or 72 h. After that, cells were rinsed once for WST-1 assay. The cells incubated with 100 μl fresh medium containing 10% WST-1 reagent for 2 h were measured by the absorbance at 450 nm. Positive controls were cells exposed to 1% Triton X-100 solution.

### In Vitro Effect of LaF_3_:Tb

9L glioma cells grown in DMEM media supplemented with 10% FBS and 100 units/ml of penicillin were seeded in 96-well plates (5000 cells/well) and cultured overnight. Then, cells received the treatment of mixed solution of LaF_3_:Tb (1 mg/ml) with MTCP (0.5 mg/ml) for 4 h (*n* = 5). Subsequently, they were washed with phosphate-buffered saline (PBS) twice and then exposed to portable X-ray systems (PX-80M, PoYe, Taiwan) for 1 min. The X-ray source was set at 10 mA and 80 kVp with 50 cm of exposed distance from generator to sample. The effect was evaluated after cell incubation for 24 h and analyzed by WST-1 assay (Roche) according to the manufacturer’s protocol. Cells treated with PBS were used as control groups. All values were presented as mean ± standard deviation (SD) in quintet repeat. Statistical analysis was performed using Student’s *t* test. Values of *p* < 0.05 were considered as statistically significant.

## Results and Discussion

### Materials Characterization

**Fig. 1:**
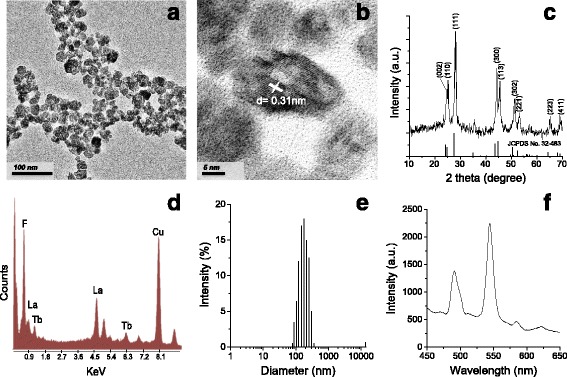
Characterization of LaF_3_:Tb particles. **a** TEM images; **b** crystal lattice planes; **c** XRD pattern and with standard data quote from JCPDS file no. 32-483; **d** EDX spectrum; **e** hydrodynamic size distribution of particles suspended in culture medium supplemented with 10% FBS; and **f** photoluminescence spectrum of particles obtained in water using an excitation wavelength of 260 nm

The study shows that the LaF_3_:Tb nanoparticles can potentially be activated by soft X-ray and used to activate PDT as a promising treatment of glioma cells. LaF_3_:Tb is formed by the self-recrystallization that the aggregative assemblies of La^3+^ and F^−^ precursors tended to form which is a crystallographic orientation under hydrothermal process. Meanwhile, the hydrothermal can lead the Tb^3+^ ions to substitute the lattice of La^3+^ in LaF_3_ crystallite [8]. TEM images revealed the particles were fabricated uniformly in size with hexagon-like shape. The size of the particles was about 25 nm with a little agglomeration (Fig. 1a). The nanopores (about 3–5 nm) were observed homogeneously distributed on the surface of particles due to the restrictions at the interface of mismatched lattices during the self-recrystallization [8]. Within a single nanoparticle, lattice spacing value was measured to be 0.31 nm, corresponding to the d-spacing of the (111) plane in the hexagonal LaF_3_ crystal (Fig. 1b). The XRD also showed the similar pattern belonging to a hexagonal structure of LaF_3_ crystals (JCPDS standard card no. 32-483), and no extra peaks were observed in the spectrum; however, peaks were slightly shifted to larger angles (Fig. 1c), referring that the particles mainly comprised Tb^3+^-doped LaF_3_ particles. The calculated lattice parameters of LaF_3_:Tb (*a* = *b* = 7.0866 nm and *c* = 7.2198 nm) were smaller than those of the LaF_3_ crystal (*a* = *b* = 7.1871 nm and *c* = 7.3501 nm), which can be attributed to the smaller radius of the Tb^3+^ ion (92.3 pm) in comparison to the La^3+^ ion (103.2 pm) [20]. Additionally, the EDX also clearly showed the composition of La, F, and Tb ions in particles, further proving the substitution of Tb^3+^ ions in LaF_3_ crystalline (Fig. 1d). Cu was detected in the spectrum because LaF_3_:Tb particles were dropped onto the TEM support film, the copper grids, under the detection of EDX.

In order to be admitted into the biomedical area, it is an important issue to take the fabrication of water-dispersible nanoparticles into consideration. In this study, LaF_3_:Tb nanoparticles can be well dispersed in aqueous solution (polydispersity index = 0.137). The hydrodynamic size of particles is approximately 157.3 nm (Fig. 1e), which is around eightfold greater than the physical diameter (Fig. 1a). The discrepancy is reasonable because it resulted from the presence of clumping and included hydration layers of water on particles when the particles are in an aqueous solution.

Fluorescence emission spectra of LaF_3_:Tb can be measured under UV or X-ray excitation, which can excite LaF_3_:Tb nanoparticles to almost the same emission peaks [14]. Upon excitation with a wavelength of 260 nm, a fluorescence of LaF_3_:Tb was clearly observed at four typical emissions peaks (480–510, 525–560, 575–590, and 615–630 nm) due to the absorption energy level of Tb^3+^ ions from 4f to 5d (Fig. 1f). The dominant green band around 540 nm can be caused by the ^5^D_4_ to ^7^F_j_ (*j* = 6–3) transitions of Tb^3+^ [21]. Overall, the results demonstrate that the LaF_3_:Tb nanoparticle could be used in biological applications and regulate photosensitizer activation by X-ray.

### In Vitro Effect of LaF_3_:Tb Nanoparticles

**Fig. 2:**
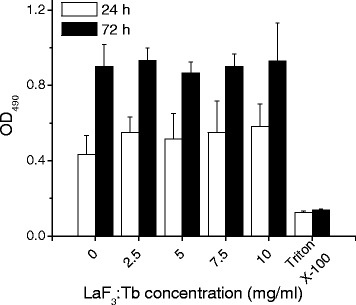
The viability of LaF_3_:Tb nanoparticles. LaF_3_:Tb nanoparticles assessed in 3T3 cells were determined using various concentrations by use of the WST-1 assay. Cells exposed to 1% Triton X-100 solution were regarded as positive control. Values are mean ± SD in triplicate

For in vitro study, the biocompatibility of LaF_3_:Tb nanoparticles is a concern. Here we assessed the effects of nanoparticles on viability of cells by use of WST-1 assay (Fig. 2). The viability of cultured fibroblast cells (3T3) to LaF_3_:Tb was determined using various concentrations. The results showed LaF_3_:Tb nanoparticles have a good biocompatibility and the cytotoxicity effect was not obviously implied as it can be seen on closer inspection within 10 mg/ml.

**Fig. 3:**
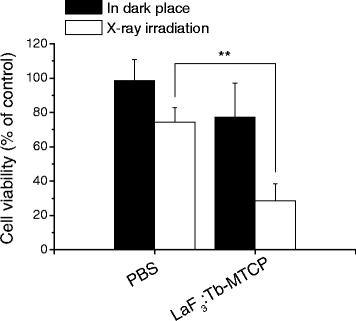
The impact of LaF_3_:Tb-MTCP nanoparticles with X-ray irradiation. The particles of LaF_3_:Tb-MTCP were assessed in 9L glioma cells. The X-ray source was set at 10 mA and 80 kVp with 50 cm of exposed distance from generator to sample. The effect was evaluated after cell incubation for 24 h and analyzed by WST-1 assay. Values are mean ± SD for quintet repeat. **p < 0.01 vs. control by Student’s *t* test

Because of the encouraging results from the viability assay, we further studied the impact of X-ray on LaF_3_:Tb nanoparticles at a concentration of 1 mg/ml with photosensitizers (LaF_3_:Tb-MTCP). In consideration of the exact spectrum match of the spectral LaF_3_:Tb’s emission and the photosensitizer’s absorption to achieve a high FRET efficiency, MTCP was chosen to be combined with LaF_3_:Tb. MTCP has demonstrated that the absorption spectrum overlaps well with the emission band (543 nm) of LaF_3_:Tb nanoparticles [11]. The results, examined in rat glioblastoma 9L cell line, showed the cell viability was decreased in the control group (PBS) when cells were exposed to soft X-ray (Fig. 3). Although it is widely accepted that malignant glioma is one of the most radioresistant tumor types, cells can be sensitive to a low radiation dose because their repair mechanisms are not induced [22]. Importantly, when LaF_3_:Tb-MTCP groups were excited by X-ray, the cell viability significantly reduced from 77 to 28% rather than the decrease in the control groups (*p* < 0.01). The cell viability of LaF_3_:Tb-MTCP in a dark place (77%) might be due to a mild toxicity caused by MTCP. However, a significant decrease of viability in LaF_3_:Tb-MTCP was mainly due to the excited LaF_3_:Tb nanoparticles because they can transfer the X-ray energy to MTCP and induce ^1^O_2_ generation to destruct the tumor, whereas the energy transfer has not been found in X-ray-excited MTCP [11]. Indeed, we may not escape the possibility of cytotoxicity (photoelectric and Compton effects) induced by X-ray on LaF_3_:Tb nanoparticles; however, this effect generally only happens in high-energy excitation (more than 500 keV) [23].

In this study, MTCP was adsorbed onto the LaF_3_:Tb surface by simply mixing the LaF_3_:Tb particles with MTCP (data not shown). It has been proved by Liu et al. [11] that MTCP can be spontaneously adsorbed onto the LaF_3_:Tb surface due to an electrostatic interaction between the positively charged LaF_3_:Tb, from unsaturated surface Tb^3+^ atoms, and the deprotonated carboxylate groups of MTCP at neutral pH [14, 24]. Although the treated solution might contain some free MTCP, the efficient energy transfer can only occur from LaF_3_:Tb nanoparticles to MTCP and induce ^1^O_2_ generation if they are situated in close proximity.

Although almost all nanoparticles do not efficiently overcome the BBB to brain tumor sites, some exceptions have been reported in recent years [25–28]. Wu et al. [26] employed SiO_2_ nanoparticles, which are 15 nm in physical diameter and 156 nm of hydrodynamic size, and the study showed that the particles can majorly accumulate in the olfactory bulb, striatum, and hippocampus through intranasal instillation. Additionally, Hirschberg’s laboratory [29, 30] has investigated the advantage of using monocytes and macrophage as cell-based delivery vehicles to ingest large payloads of nanoparticles such as gold nanoparticles or superparamagnetic iron oxide nanoparticles. Thus, we believe LaF_3_:Tb nanoparticles in the future with appropriate design or delivering route can efficiently overcome the BBB for non-invasive PDT in brain cancer treatment.

## Conclusions

According to our preliminary finding, LaF_3_:Tb nanoparticles could find biological applications, for they have been obtained in nanoscale (approximately 25 nm in physical size), water-dispersible, and with high biocompatibility. However, it shows cytotoxicity on the 9L glioma cell line only when nanoparticles with photosensitizers are exposed under the X-ray exposure. Thus, we believe scintillating nanoparticles in combination with X-ray could be a potential approach for non-invasive PDT in brain cancer for future clinical applications, even though an ideal scintillating nanoparticle that processes the energy transfer from X-ray to photosensitizers efficiently will still be an important issue for practical applications. We will further investigate the in vivo study in the following research.

## Abbreviations

BBB: Blood-brain barrier
DMEM: Dulbecco’s modified Eagle’s medium
EDX: Energy-dispersive X-ray spectroscopy
FBS: Fetal bovine serum
FRET: Fluorescence resonance energy transfer
LaF_3_:Tb: Tb^3+^-doped LaF_3_ scintillating nanoparticles
MTCP: Meso-tetra(4-carboxyphenyl)porphyrin
PBS: Phosphate-buffered saline
PDT: Photodynamic therapy
SD: Standard deviation
TEM: Transmission electron microscopy
WST-1: Cell proliferation reagent
XRD: X-ray diffraction
